# Adaptive Kalman Filter-Based UWB Location Tracking with Optimized DS-TWR in Workshop Non-Line-of-Sight Environments

**DOI:** 10.3390/s25247682

**Published:** 2025-12-18

**Authors:** Jian Wu, Yijing Xiong, Wenyang Li, Wenwei Xia

**Affiliations:** College of Mechanical Engineering, Nanjing University of Science and Technology, Nanjing 210094, China; 13986966749@163.com (Y.X.); 13677267386@163.com (W.L.); 15601590859@163.com (W.X.)

**Keywords:** UWB, DS-TWR, adaptive Kalman filter (AKF), non-line-of-sight workshop environment

## Abstract

At the current stage, indoor Ultra-Wideband (UWB) positioning systems often encounter challenges in achieving high localization accuracy under non-line-of-sight (NLOS) conditions within workshop environments when employing the Double-Sided Two-Way Ranging (DS-TWR) algorithm. To address this issue, a positioning optimization method based on the DS-TWR algorithm is proposed. By streamlining message exchanges between nodes, the method reduces node energy consumption and shortens ranging time, thereby enhancing system energy efficiency and response speed. Furthermore, to improve positioning accuracy in workshop NLOS environments, an Adaptive Kalman Filtering algorithm is introduced. This algorithm dynamically evaluates the influence of obstruction information caused by NLOS conditions on the covariance of observation noise and adaptively adjusts the filtering gain of the signals accordingly. Through this approach, the system can effectively eliminate invalid positioning information in signals, mitigate the adverse effects of NLOS conditions on positioning accuracy and achieve more precise localization. Experimental results demonstrate that the proposed optimization algorithm achieves substantial performance improvements in both static and dynamic positioning experiments under workshop NLOS conditions. Specifically, the algorithm not only enhances system positioning accuracy but also further strengthens the real-time ranging precision of the DS-TWR algorithm.

## 1. Introduction

With the rapid advancement of technology, the dependence of intelligent and information-based services on location information has shown a significant upward trend in contemporary society. Against this backdrop, wireless positioning technology, owing to its unique advantages, has assumed a pivotal role in the digitalization of industrial operations. Specifically, wireless positioning plays a crucial role in enabling precise localization within production workshops, thereby not only driving transformation in the industrial sector but also effectively improving production efficiency [1]. In the current field of indoor positioning technologies, multiple approaches such as ZigBee, Wi-Fi, and LiDAR [2] each exhibit distinctive advantages; however, their positioning accuracy in complex workshop environments remains generally limited. With the deepening of intelligent manufacturing, the demand for higher positioning accuracy has continued to increase. In this context, Ultra-Wideband (UWB) technology has attracted considerable attention due to its unique performance characteristics. UWB [3], with its strong anti-interference capability, high transmission rate, and exceptional time resolution, demonstrates significantly superior indoor positioning accuracy compared with traditional narrowband communication technologies such as Wi-Fi and Bluetooth. For instance, the DW1000 chip developed by DecaWave achieves a ranging accuracy of up to 10 cm [4]. Moreover, UWB exhibits strong resistance to multipath effects and robust adaptability to non-line-of-sight (NLOS) conditions, which renders it particularly advantageous in workshop environments that contain numerous obstacles such as glass, metal, and concrete [5,6].

Given the complexity of the workshop environment, this paper adopts an optimized Double-Sided Two-Way Ranging (DS-TWR) algorithm to perform high-precision distance measurement in industrial workshops. To handle non-line-of-sight (NLOS) conditions, an adaptive Kalman filtering algorithm is further introduced to post-process the ranging data obtained by the DS-TWR algorithm.

In addition, compared with previous NLOS-mitigation methods such as IPSO-IAUKF [7] and FCN-Attention [8], the approach adopted in this paper follows a different design philosophy. IPSO-IAUKF mainly relies on multi-sensor fusion (UWB + IMU) and an optimization process to adjust the parameters of an interacting adaptive UKF, which inevitably increases system complexity and computational cost. FCN-Attention, on the other hand, employs a deep-learning-based LOS/NLOS classifier that requires large amounts of labeled data and additional computational resources for network training and inference. In contrast, our work focuses on improving the DS-TWR ranging procedure itself and introduces a lightweight covariance-based adaptive Kalman filter that operates directly on the ranging results. This design eliminates the need for extra sensors or offline training, while still effectively suppressing NLOS-induced measurement fluctuations in workshop environments. Therefore, the novelty of this paper lies in combining an optimized DS-TWR ranging protocol with a covariance-driven adaptive Kalman filter specifically designed for DS-TWR measurements under NLOS conditions, providing a simple and practical solution for resource-constrained industrial workshops.

## 2. DS-TWR Ranging Algorithm

### 2.1. Principle of DS-TWR Ranging

**Figure 1:**
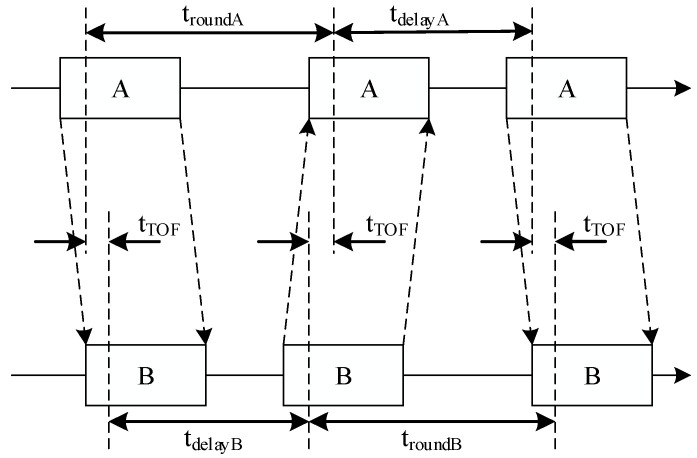
Double-sided two-way ranging principle.

The DS-TWR ranging algorithm determines the time of flight (ToF) between the anchor and the tag by recording two round-trip timestamps [9,10,11]. The detailed procedure of the Double-Sided Two-Way Ranging (DS-TWR) algorithm is illustrated in Figure 1. First, a message frame is transmitted from node A to node B and reaches node B after a propagation delay. Upon reception, a confirmation message frame is transmitted back to node A following a response delay. After receiving the confirmation message frame from node B, node A, after another response delay, transmits a final message frame to node B [12].

Ultimately, the signal transmission time is derived from Equation (1) and subsequently converted into distance.

(1) $$t_{TOF}=\frac{\left(t_{roundA}\times t_{roundB}-t_{delayA}\times t_{delayA}\right)}{\left(t_{roundA}+t_{roundB}+t_{delayA}+t_{delayA}\right)}$$

Once the time of flight $t_{TOF}$ is calculated, it is multiplied by the speed of light constant to yield the distance value, completing the TWR ranging procedure. In the subsequent equation, C represents the speed of light constant, which is taken as 3.0 × 10^8^ m/s:

(2) $$S=t_{TOF}\times C$$

### 2.2. Optimized DS-TWR Ranging Algorithm

Considering that the presence of personnel, machinery, and other facilities in a workshop may obstruct UWB signals and cause non-line-of-sight (NLOS) errors, accurate ranging and positioning in such environments typically require four anchors to measure simultaneously [13]. According to this method, the tag needs to send eight messages, while the anchors send four messages, resulting in a total of twelve messages. Frequent message exchanges increase power consumption and ranging time. To address this, we employ an optimized DS-TWR ranging algorithm. With a configuration of one tag and four anchors, the ranging procedure can be completed with only six message exchanges (two transmitted by the tag and four by the anchors), thereby reducing both communication latency and node power consumption [14]. For clarity of message categorization, the first transmission from the tag is designated as the Poll message, the responses from the four anchors are defined as RS messages, and the final transmission from the tag is defined as the FINAL message [15,16].

**Figure 2:**
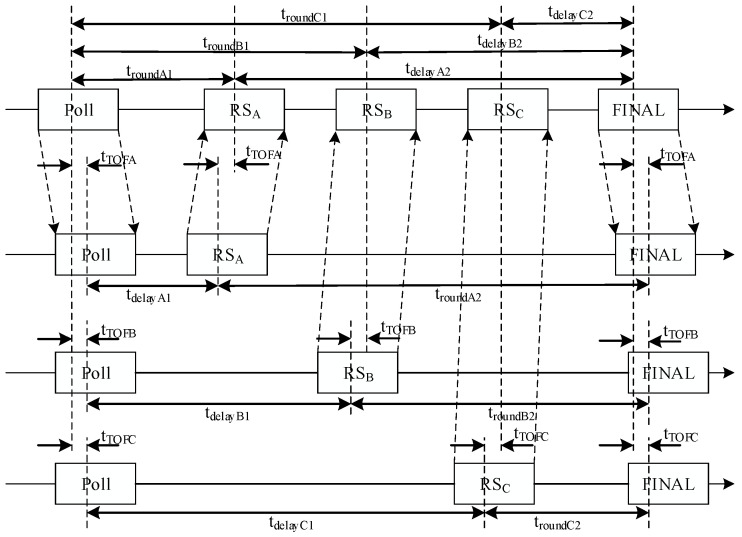
Optimized double-sided two-way ranging principle.

As illustrated in Figure 2, the optimized DS-TWR algorithm performs a ranging cycle in which the tag first transmits a Poll message, the four anchors (A, B, C, and D) sequentially respond with RS messages, and finally, the tag sends a FINAL message. In contrast to the conventional TWR algorithm, where one ranging operation is completed with a single exchange of Poll, RS, and FINAL messages, the optimized DS-TWR algorithm significantly reduces the number of message transmissions, thereby lowering node power consumption and shortening the ranging duration.

According to Figure 2, the time of flight (ToF) from the tag to Anchor A is

(3) $$t_{TOFA}=\frac{t_{roundA1}\times t_{roundA2}-t_{delayA1}\times t_{delayA2}}{t_{roundA1}+t_{roundA2}+t_{delayA1}+t_{delayA2}}$$

Similarly, the time of flight (ToF) from the tag to Anchors B and C can be obtained as

(4) $$t_{TOFB}=\frac{t_{roundB1}\times t_{roundB2}-t_{delayB1}\times t_{delayB2}}{t_{roundB1}+t_{roundB2}+t_{delayB1}+t_{delayB2}}$$

(5) $$t_{TOFC}=\frac{t_{roundC1}\times t_{roundC2}-t_{delayC1}\times t_{delayC2}}{t_{roundC1}+t_{roundC2}+t_{delayC1}+t_{delayC2}}$$

**Figure 3:**
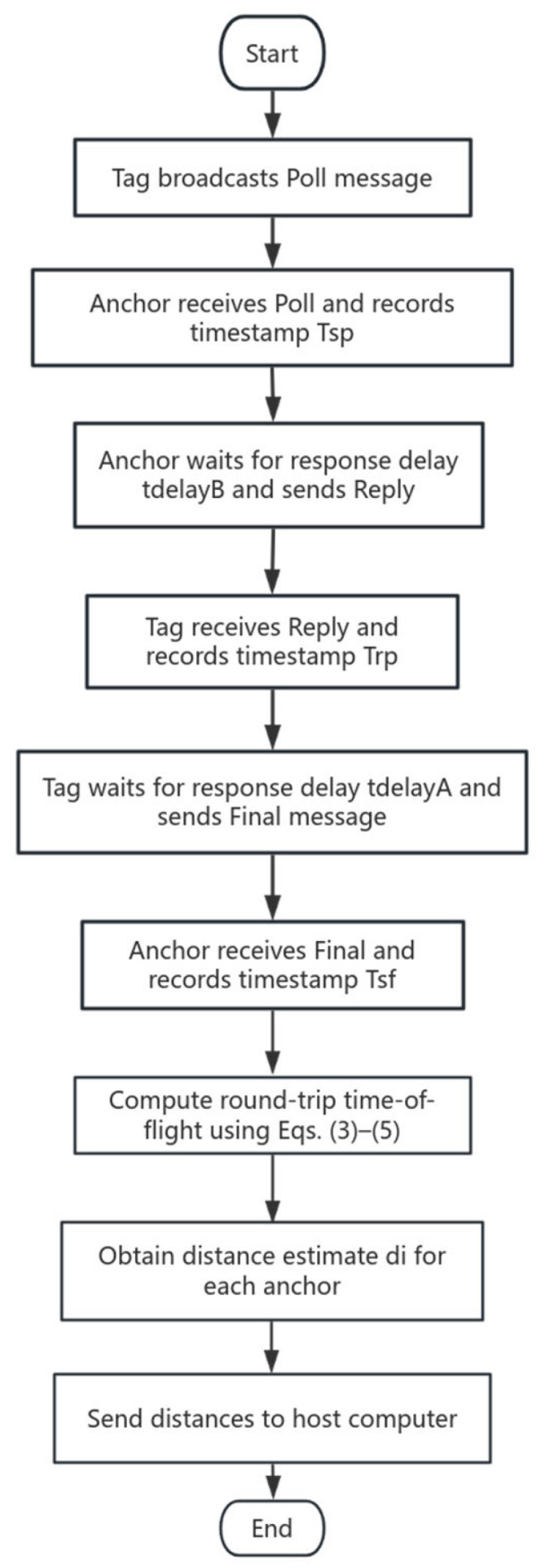
Flowchart of the optimized DS-TWR ranging algorithm.

The overall processing procedure of the optimized DS-TWR ranging algorithm is summarized in the flowchart shown in Figure 3. Starting from the tag, a Poll message is transmitted to the anchors, and each anchor responds after its response delay t*_delayB_*. The tag also applies a response delay t*_delayA_* before sending the Final message. All required timestamps are recorded during this process, and the round-trip time-of-flight is then computed using Equations (3)–(5).

## 3. Adaptive Kalman Filter (AKF) Algorithm

### 3.1. Principle of Improved Adaptive Kalman Filter Algorithm

In theoretical analysis, with the increasing number of iterations, the error covariance matrix of the Kalman filter algorithm typically converges gradually to a steady state. However, this theoretical assumption faces significant challenges in practical workshop environments. In complex workshop settings, moving machinery and personnel often block UWB signals, causing multipath and NLOS errors [17] that degrade positioning accuracy. To address this issue, this paper proposes an improved adaptive Kalman filter algorithm. By adaptively adjusting the filtering gain, the algorithm effectively eliminates invalid positioning information in UWB signal [18,19]. This approach significantly enhances the positioning accuracy of the DS-TWR algorithm in complex workshop environments. The optimized DS-TWR scheme is first used to obtain the distance measurements, and the proposed adaptive Kalman filter is then applied to these measurements as a post-processing step to improve accuracy under NLOS conditions, without changing the underlying DS-TWR ranging procedure or the packet-exchange sequence between the tag and the anchors.

Let the extracted UWB positioning signal be ${\hat{x}}_{k}$ and the corresponding positioning result be $p_{k}$:

(6) $${\hat{x}}_{k}=a_{t-1}{\hat{x}}_{t-1}+{\hat{a}}_{t-1}$$

(7) $$p_{k}=g_{t}x_{t}+u_{t}$$

At the beginning of the filtering process, the state estimate is initialized by setting ${\hat{x}}_{0}$ to the first available ranging measurement, and the dynamic noise state ${\hat{a}}_{0}$ is initialized to zero. The initial noise-variance parameters and $W_{0}$ are chosen as small positive scalars based on the variance of static ranging data collected in the workshop environment, and these values remain fixed for all subsequent experiments.

When filtering the signal, two key state variables are defined: ${\hat{x}}_{t-1}$, denoting the state of the positioning signal, and ${\hat{a}}_{t-1}$, describing the dynamic noise state induced by obstructions. In addition, three parameters are introduced: $g_{t}$, the filtering gain used to adjust the weighting during signal processing; $u_{t}$, the noise effect on signal observations caused by obstructions; and $a_{t-1}$, the dynamic noise term mentioned above. These variables and parameters jointly influence the accuracy and performance of the UWB positioning system.

In common scenarios, dynamic noise and observation noise typically follow Gaussian distributions. The variation of a scalar noise-intensity parameter W, which characterizes these noises, directly reflects the magnitude of positioning errors in the localization system. To enhance positioning accuracy under non-line-of-sight (NLOS) workshop environments, this paper adopts a dynamic estimation method for W. The proposed approach adaptively adjusts the filtering of invalid positioning information, thereby improving the overall performance of the localization system [20].

The dynamic estimation equation of W at time t is given by

(8) $$W_{t}=M_{t-1}^{2}W_{t-1}+\mathrm{var}(\sigma_{t})$$

In this equation, $\mathrm{var}(\sigma_{t})$ represents the variance of a zero-mean Gaussian white noise sequence, which is used to characterize the dispersion of the positioning signal under random noise. $M_{t-1}^{2}$ is a complex coefficient reflecting the dynamic noise state of the signal caused by obstructions. $W_{t-1}$ denotes a scalar measure of the observation-noise intensity at time t−1, describing the magnitude of the observation residual.

**Figure 4:**
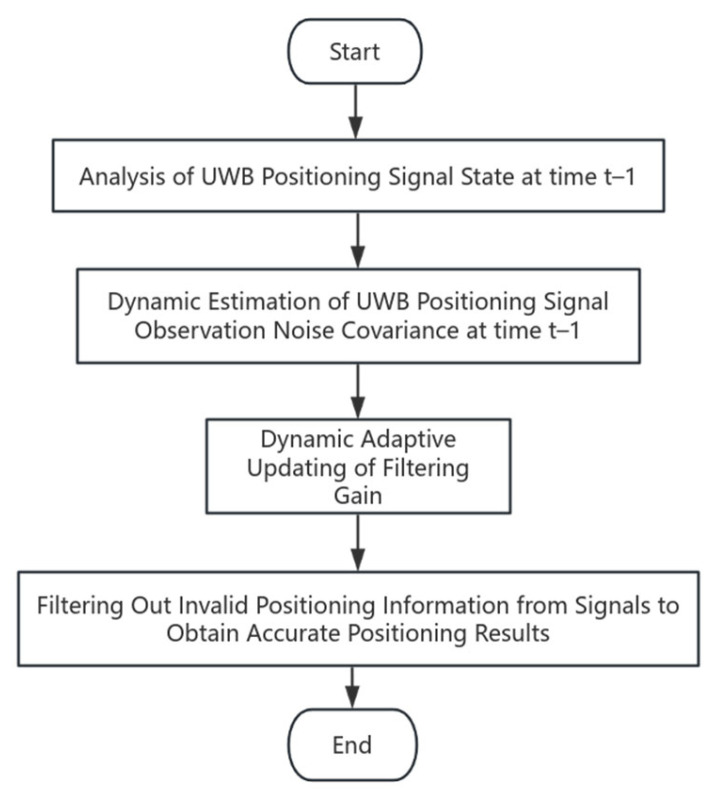
Flowchart of the adaptive Kalman filtering algorithm.

The flowchart of the adaptive Kalman filtering algorithm is shown in Figure 4.

Accordingly, to improve the positioning accuracy within the workshop environment at time t, the adjustment equation of the filtering gain $g_{t}$ is given by

(9) $$g_{t}=(P_{t}W_{t}+P_{t})\times (W_{t-1}P_{t-1}W_{t-1}^{T}+P_{1}+W_{t-1})$$

Here, $P_{t}$ and $W_{t}$ denote the scalar noise-variance parameters of the dynamic noise and observation noise at time t, respectively. These two parameters are used to quantify the characteristics of the noise. The superscript T represents the transpose operation.

The adjusted filtering gain $g_{t}$, obtained from Equation (9), is substituted into Equation (7) and the invalid positioning information in the signal can be effectively eliminated, thereby yielding more accurate localization results.

**Figure 5:**
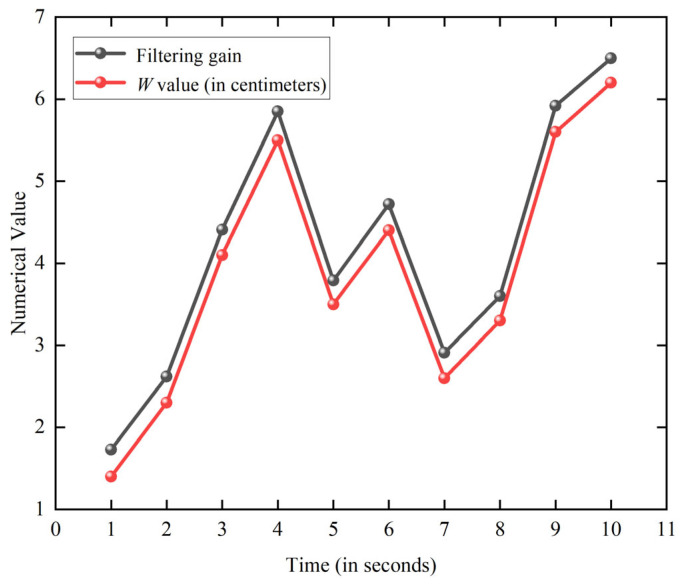
Adaptive Kalman filter gain adjustment results.

The simulation results of the proposed algorithm, obtained through MATLAB R2020b simulations, are presented in Figure 5.

As illustrated in Figure 5, the adaptive Kalman filter algorithm is capable of dynamically estimating W and adaptively adjusting the filtering gain while ensuring that the adjusted gain remains greater than W. Here, W is a time-varying scalar parameter that quantifies the intensity of the observation noise in the UWB measurements, as defined in Equation (8). A larger W indicates stronger measurement uncertainty (e.g., NLOS effects), whereas a smaller W corresponds to reliable LOS measurements. When the filtering gain is greater than W, the filter relies more on the current measurement, which is desirable under low-noise conditions. When W increases, the gain decreases accordingly, preventing the filter from overreacting to high-noise measurements. This adaptive mechanism effectively enhances the positioning accuracy.

### 3.2. Localization Algorithm

Based on the filtered ranging results, the final tag position is computed using a two-dimensional trilateration method. A Cartesian coordinate system is established in the workshop, and the coordinates of the N anchors are represented as $\left(x_{i},y_{i}\right)$. After DS-TWR ranging and adaptive Kalman filtering, the estimated distance between the tag and the i-th anchor is denoted as $d_{i}$. Ideally, the tag position (x,y) satisfies the geometric constraint ${\left(x-x_{i}\right)}^{2}+{\left(y-y_{i}\right)}^{2}=d_{i}^{2}$. To obtain a computationally efficient solution, these nonlinear equations are linearized by subtracting the equation of a reference anchor from the others, generating a set of linear equations in the form Ap=b, where $p=\left[x\right.\;{\left.y\right]}^{T}$ is the unknown position vector. The least-squares estimate of the tag position is then obtained by $p={(A^{T}A)}^{-1}A^{T}b$. This trilateration-based localization algorithm is executed on the host computer following the adaptive Kalman filtering stage.

## 4. Experiments and Evaluations

### 4.1. Experimental Environment Setup

In the developed UWB positioning system, one mobile tag and four fixed anchors were deployed. Each node (tag and anchors) integrates an STM32F103C8T6 microcontroller (STMicroelectronics, Geneva, Switzerland) and a DWM1000 UWB transceiver module (Qorvo Inc., Greensboro, NC, USA), forming a unified hardware platform for both ranging and positioning tasks. The STM32F103C8T6 configures and controls the DWM1000 through an SPI interface. Anchor 1 additionally incorporates a USB-to-serial interface to upload ranging data to the host computer in real time, where the proposed adaptive Kalman filtering and positioning algorithms are executed.

The DWM1000 module used in this work complies with the IEEE 802.15.4-2011 UWB physical-layer specification and is designed for high-precision ranging and low-power wireless communication [21,22]. In our implementation, all nodes share the same UWB physical-layer parameter configuration, including RF channel, center frequency, pulse repetition frequency, and data rate, and these parameters remain unchanged throughout all experiments. The DS-TWR ranging cycle and the corresponding position-update rate are fixed in the firmware so that all algorithms are evaluated under identical temporal sampling conditions.

**Figure 6:**
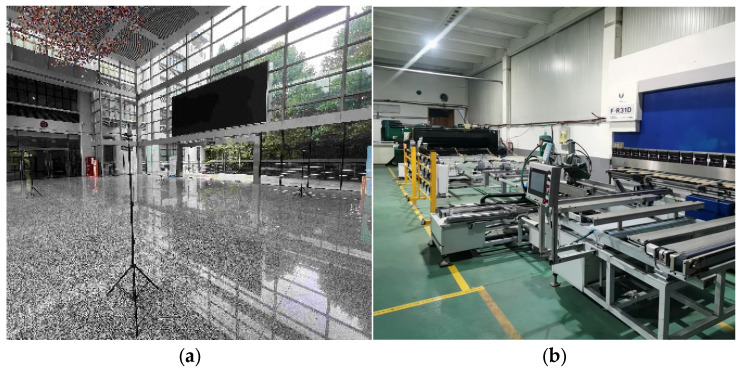
Illustration of the experimental environments: (**a**) Indoor LOS environment; (**b**) Workshop NLOS environment.

For both the point-to-point ranging experiment and the adaptive Kalman filtering–based ranging and positioning experiment under line-of-sight (LOS) indoor conditions, the experimental environment was the first-floor hall of the Engineering Training Center at Nanjing University of Science and Technology. For the non-line-of-sight (NLOS) scenario, the experiments were conducted in a mechanical workshop of the same training center. The experimental setups are illustrated in Figure 6.

**Figure 7:**
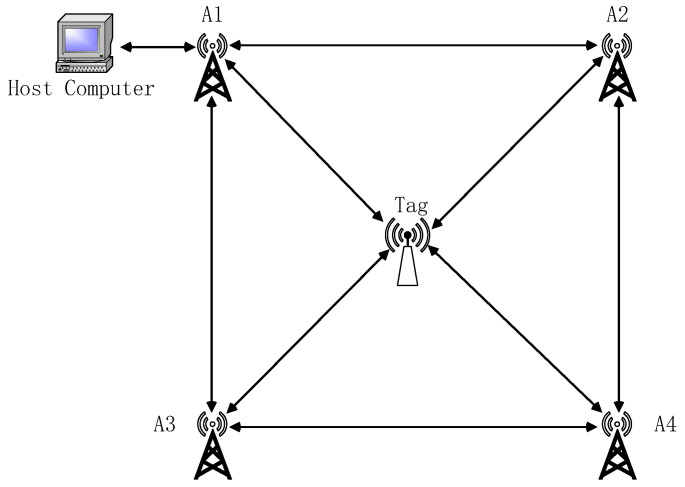
Schematic diagram of node arrangement.

In the dynamic positioning experiment based on the adaptive Kalman filtering algorithm, the experimental area was set to 700 cm × 700 cm. A Cartesian coordinate system was established with Anchor 1 as the origin, and the coordinates of the remaining anchors were determined accordingly. To ensure that at least three anchors could simultaneously perform ranging with the tag under non-line-of-sight (NLOS) conditions, four anchors were deployed for the dynamic positioning experiment. The layout of all nodes is shown in Figure 7.

In the dynamic positioning experiment, the coordinates of Anchor 1 were set as the origin (0, 0), while Anchors 2, 3, and 4 were located at (0, 700), (700, 0), and (700, 700), respectively. The tag was initialized at the starting point (200, 200) and sequentially passed through points A, B, and C before returning to the origin. The coordinates of points A, B, and C were (500, 200), (500, 500), and (200, 500), respectively. The overall trajectory of the tag formed a square with a side length of 300 cm. All coordinates are expressed in centimeters.

### 4.2. Point-to-Point Ranging Experiment

**Figure 8:**
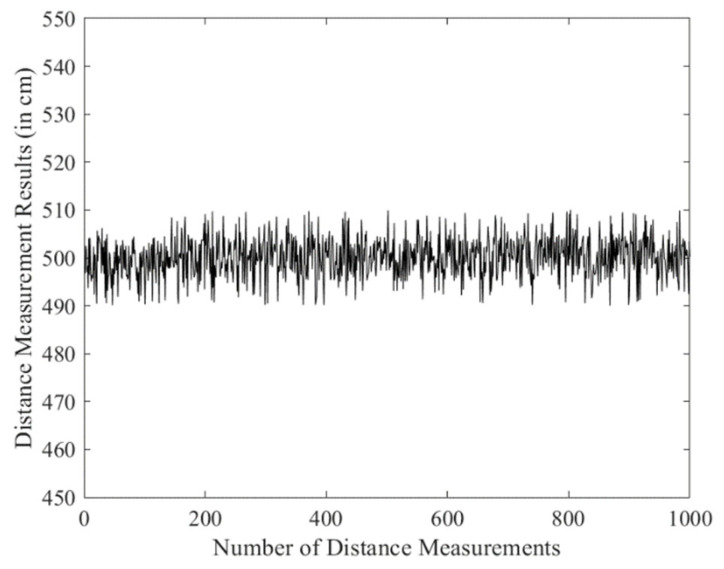
500 cm ranging experiment.

**Figure 9:**
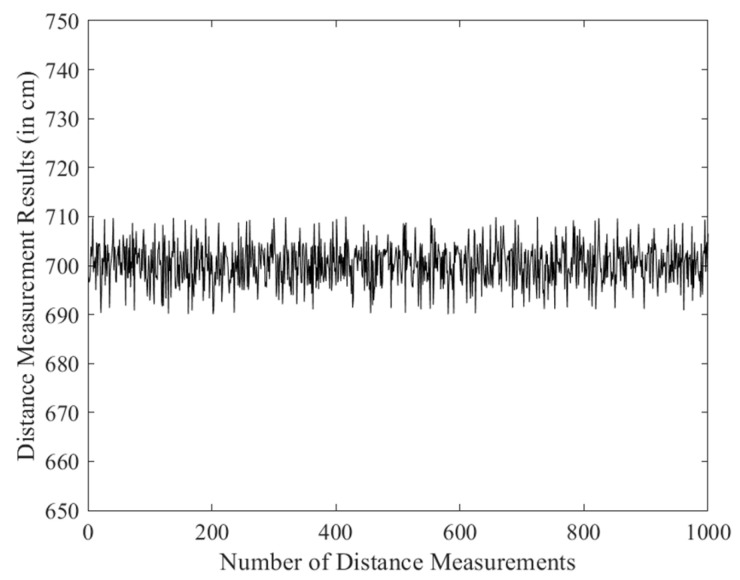
700 cm ranging experiment.

**Figure 10:**
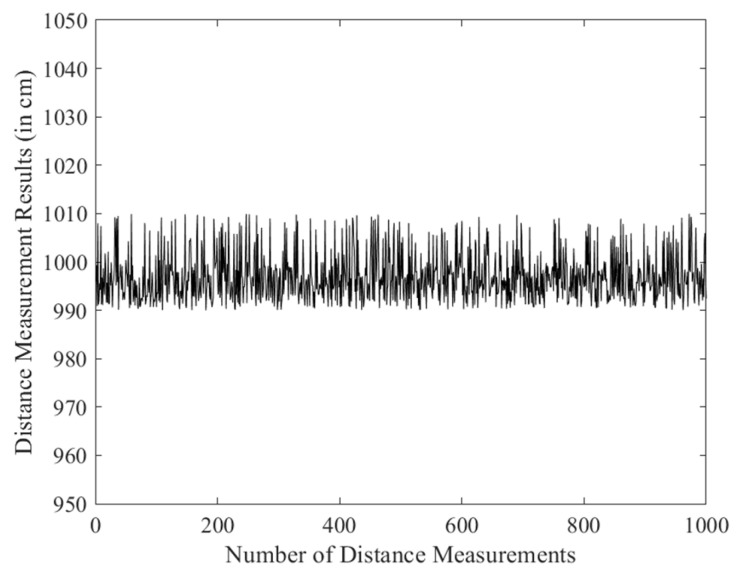
1000 cm ranging experiment.

In the distance measurement experiment, a fixed-point testing method was adopted, where the anchors were kept stationary while the tag was moved. The tag was placed at distances of 500 cm, 700 cm, and 1000 cm from the anchor to conduct the ranging tests. For each position, 1000 distance measurements were collected, and the data were exported to generate line plots, as shown in Figure 8, Figure 9 and Figure 10.

The distance measurements at different positions (500 cm, 700 cm, and 1000 cm) were analyzed to evaluate the accuracy and precision of the DS-TWR algorithm. The statistical values, including range, standard deviation (σ), and mean absolute deviation (MAD) for each measurement distance, are summarized in Table 1.

The results show that the ranging accuracy can reach 10 cm, and the collected distance data exhibited good stability. These findings further verify the reliability and effectiveness of the optimized DS-TWR ranging algorithm.

### 4.3. Adaptive Kalman Filtering–Based Ranging and Positioning Experiment

Ranging experiments were conducted under three conditions: without Kalman filtering, with standard Kalman filtering, and with adaptive Kalman filtering. The experiments were performed in two scenarios, namely indoor line-of-sight (LOS) and workshop non-line-of-sight (NLOS) environments, to enable performance comparison. Quantitative analysis was carried out using mean absolute error (MAE), mean squared error (MSE), and root mean squared error (RMSE) [23]. The results, calculated to three decimal places, are presented in Table 2 and Table 3.

An analysis of the data in Table 2 and Table 3 shows that, under indoor line-of-sight (LOS) conditions, the performance of the standard Kalman filtering algorithm and the adaptive Kalman filtering algorithm in ranging is comparable. Both approaches significantly improve ranging accuracy compared with the case without Kalman filtering, and the results are closer to the ground truth. However, in the more complex workshop non-line-of-sight (NLOS) environment, the error of the standard Kalman filter increases markedly, indicating its poor adaptability to NLOS conditions. In contrast, the adaptive Kalman filtering algorithm maintains a relatively stable error level under NLOS conditions, with performance that is not substantially different from that under LOS conditions. In workshop NLOS scenarios, the adaptive Kalman filter reduces ranging error by about 54%, confirming its superior accuracy and practicality.

### 4.4. Adaptive Kalman Filtering–Based Dynamic Positioning Experiment

**Figure 11:**
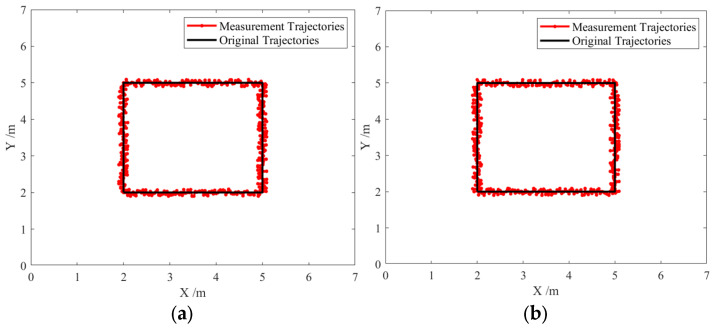
Comparison of localization trajectories under line-of-sight (LOS) conditions: (**a**) Trajectory of the improved algorithm; (**b**) Trajectory of the original algorithm.

**Figure 12:**
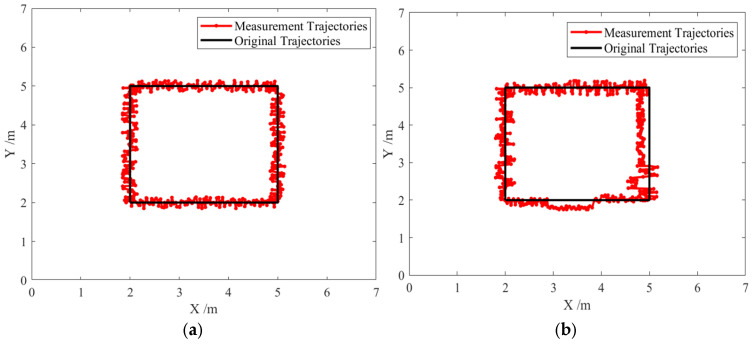
Comparison of localization trajectories in the workshop under non-line-of-sight (NLOS) conditions: (**a**) Trajectory of the improved algorithm; (**b**) Trajectory of the original algorithm.

To simulate a practical scenario in which a user carries a positioning tag while performing tasks, the experimenter carried the tag and followed a predefined trajectory in the test area at a normal, approximately constant walking speed representative of typical movement in a workshop. Since it is difficult to obtain the exact real-time position of the tag during movement, trajectory consistency was adopted as the evaluation criterion. First, a motion trajectory was defined in the test environment, and the experimenter moved along the predefined path. The collected data from the moving tag were then processed to reconstruct the estimated trajectory, which was compared with the ground-truth trajectory. The experimental environment was the same as that described in Section 4.1. Both the adaptive Kalman filtering algorithm and the standard Kalman filtering algorithm were applied for data filtering, followed by trilateration for positioning [24,25], and the resulting trajectories are compared in Figure 11 and Figure 12.

Figure 11 and Figure 12 show that under LOS conditions, both algorithms achieve similar performance with errors below 10 cm and trajectories close to the ground truth. However, when the environment shifts to workshop non-line-of-sight (NLOS) conditions, a significant difference emerges between the two algorithms. The standard Kalman filter exhibits a substantial increase in positioning error, reaching up to 45 cm, and its dynamic trajectory deviates considerably from the true path. In contrast, the adaptive Kalman filtering algorithm demonstrates higher stability and accuracy, maintaining positioning errors within 15 cm, with its dynamic trajectory remaining closely aligned with the original path. These results indicate that, in workshop NLOS environments, the adaptive Kalman filtering algorithm achieves superior positioning performance with higher accuracy.

#### Statistical Reliability and Real-Time Performance

It should be noted that the dynamic experiment contains several hundred consecutive UWB ranging and positioning measurements, which naturally form a sufficiently large statistical sample. Therefore, instead of performing multiple independent experimental repetitions, the statistical characteristics can be analyzed directly from the complete time series of measurements in each experiment. This approach provides meaningful statistical reliability without modifying the experimental setup.

From the dynamic time series, it can be observed that the error distribution remains stable throughout the experiment and no abrupt divergence occurs. This indicates that the adaptive Kalman filtering algorithm maintains consistent performance over time, and the observed error characteristics are representative and statistically reliable.

In addition to positioning accuracy, the proposed method is computationally lightweight in practice. In our implementation, the complete processing pipeline, including DS-TWR ranging, adaptive Kalman filtering, and position estimation, runs in real time on a standard PC without noticeable latency during the dynamic experiments, which confirms that the proposed scheme meets the real-time requirements of typical industrial UWB positioning applications.

## 5. Conclusions

This paper addresses the challenges of ultra-wideband (UWB) indoor positioning systems in workshop non-line-of-sight (NLOS) environments by proposing an optimized DS-TWR algorithm and introducing an adaptive Kalman filtering approach to enhance positioning accuracy and overall system performance. Based on the experimental results, the following conclusions are drawn:

1. By simplifying the message exchange process between nodes, the optimized DS-TWR algorithm effectively reduces node energy consumption and shortens ranging time, thereby improving system energy efficiency and response speed.
2. Under NLOS conditions, the adaptive Kalman filtering algorithm is capable of dynamically evaluating the impact of obstruction on the observation noise covariance and adaptively adjusting the filter gain [26]. This mechanism effectively eliminates invalid positioning information and significantly improves positioning accuracy.
3. Experimental results in both indoor LOS and workshop NLOS environments demonstrate that the proposed optimization methods exhibit stable performance in both static and dynamic positioning, verifying their practical applicability and potential for deployment in industrial environments.

## Figures and Tables

**Table 1:** Statistical Analysis of Distance Measurements.

Distance (cm)	Rang (cm)	Standard Deviation (σ)	MAD
500	19	5.021	4.693
700	19	3.986	3.075
1000	20	5.529	4.458

**Table 2:** Ranging error analysis for indoor line-of-sight case.

Distance Measurement Algorithm	MAE	MSE	RMSE
No filtering	7.927	87.534	15.467
Standard Kalman filtering	4.032	17.658	4.373
Adaptive Kalman filtering	3.409	15.823	4.195

**Table 3:** Ranging error analysis for non-line-of-sight cases in the workshop.

Distance Measurement Algorithm	MAE	MSE	RMSE
No filtering	14.982	125.278	17.421
Standard Kalman filtering	8.294	34.523	9.354
Adaptive Kalman filtering	3.783	16.231	3.925

## Data Availability

The original contributions presented in this study are included in the article. Further inquiries can be directed to the corresponding author.

## Abbreviations

UWB	Ultra-Wideband
DS-TWR	Double-Sided Two-Way Ranging
LOS	Line-of-Sight
NLOS	Non-Line-of-Sight
AKF	Adaptive Kalman Filter
ToF	Time of Flight
MAD	mean absolute deviation
MAE	Mean Absolute Error
MSE	Mean Squared Error
RMSE	Root Mean Square Error
